# Identification of Factors Influencing the Restoration of Cyanobacteria-Dominated Biological Soil Crusts

**DOI:** 10.1371/journal.pone.0090049

**Published:** 2014-03-13

**Authors:** Chongfeng Bu, Shufang Wu, Yongsheng Yang, Mingguo Zheng

**Affiliations:** 1 Institute of Soil and Water Conservation, Northwest A & F University, Yangling, Shaanxi, China; 2 Institute of Soil and Water Conservation, Chinese Academy of Sciences and Ministry of Water Resources, Yangling, Shaanxi, China; 3 College of Water Resources and Architectural Engineering, Northwest A & F University, Yangling, Shaanxi, China; 4 Institute of Geographic Sciences and Natural Resources Research, Chinese Academy of Sciences, Beijing, China; University of New South Wales, Australia

## Abstract

Biological soil crusts (BSCs) cover >35% of the Earth’s land area and contribute to important ecological functions in arid and semiarid ecosystems, including erosion reduction, hydrological cycling, and nutrient cycling. Artificial rapid cultivation of BSCs can provide a novel alternative to traditional biological methods for controlling soil and water loss such as the planting of trees, shrubs, and grasses. At present, little is known regarding the cultivation of BSCs in the field due to lack of knowledge regarding the influencing factors that control BSCs growth. Thus, we determined the effects of various environmental factors (shade; watering; N, P, K, and Ca concentrations) on the growth of cyanobacteria-dominated BSCs from the Sonoran Desert in the southwestern United States. The soil surface changes and chlorophyll *a* concentrations were used as proxies of BSC growth and development. After 4 months, five factors were found to impact BSC growth with the following order of importance: NH_4_NO_3_ ≈ watering frequency>shading>CaCO_3_ ≈ KH_2_PO_4_. The soil water content was the primary positive factor affecting BSC growth, and BSCs that were watered every 5 days harbored greater biomass than those watered every 10 days. Groups that received NH_4_NO_3_ consistently exhibited poor growth, suggesting that fixed N amendment may suppress BSC growth. The effect of shading on the BSC biomass was inconsistent and depended on many factors including the soil water content and availability of nutrients. KH_2_PO_4_ and CaCO_3_ had nonsignificant effects on BSC growth. Collectively, our results indicate that the rapid restoration of BSCs can be controlled and realized by artificial “broadcasting” cultivation through the optimization of environmental factors.

## Introduction

Biological soil crusts (BSCs) are highly complex communities composed of mosses, cyanobacteria, lichens, bacteria, and fungi with soil particles [1], [2]. BSCs, which are widely distributed in arid and semiarid areas of hot and cold zones, impact biogeochemical cycles and hydrological processes and function to prevent erosion [3], [4], [5], [6]. BSCs are known to enrich soil nutrients, enhance soil stability, and reduce soil erosion by wind and water [7], . For example, in the Loess Plateau, moss-dominated BSC were shown to reduce water runoff and sediment loss from soils by >30% and >80%, respectively [10]. In a separate wind tunnel experiment, moss- and cyanobacteria-dominated BSCs reduced the wind erosion rate of soils by more than 90% as compared to uncovered desert soils [11]. Therefore, attempts have been made to establish artificial BSCs to promote soil stability and to reduce losses due to wind and water erosion. Wei [12] proposed the concept of BSC carpet engineering, i.e., the creation of artificial BSCs to control sand movement. Subsequent research on BSC inoculation has progressed rapidly, especially regarding the use of artificial cyanobacteria-dominated BSC cultures. For instance, in a 200-ha experimental soil plot established in the Hobq Desert [13], [14], [15], an artificial cyanobacterial crust enhanced the activity of soil communities and accelerated soil development [16]. Moreover, the average thickness of this artificial BSC ranged from 2.23 to 5.36 mm, with coverage of 70%, after 3 years of growth, suggesting that this approach may have long-term positive effects on soil stability [17].

BSCs are generally classified into moss, lichen, and cyanobacterial crusts based on their predominant compositions. Mosses with stems, leaves, and rhizoids are considered the higher plant and have relatively strong photosynthetic capacities. Cyanobacteria, which usually act as the pioneer organisms for desert soils, are considered the lower plant characterized by low photosynthesis. Lichens are complexes of algal cells enveloped within epiphyte mycelia and can endure extremely arid environments [18]. These three organisms are characterized by different physiological properties and require multifarious environments and conditions for rapid growth, which results in the complexity of artificial restoration for each type of BSC. The influences affecting the development of BSCs can be divided into internal and external factors. Internal factors include the physiological characteristics of the species present in the BSCs and the interactions between these species, whereas external factors involve soil moisture, light intensity, temperature, and the availability of nutrients. A previous study [19] showed that the production of biomass and exopolysaccharides (EPS) by *Microcoleus vaginatus* Gom. was not linearly related to temperature, light, or the availability of nutrients. Li et al. [20] found that the colonization of cyanobacteria and algae in the earliest successional stages of BSCs was facilitated by a higher soil pH and total potassium content in the topsoil. Recent research [21] showed that BSCs amended with composted sewage sludge promoted N and C fixation and increased chlorophyll *a*. However, the frequent watering of these BSCs led to a lower diversity of cyanobacteria populations. In addition, the organic matter content, soil water content, total N, total P, bioavailable P, bioavailable K, pH, electrical conductivity, and total salt content also significantly affected the abundance of microorganisms in BSCs [22]. External factors, such as those described above, are likely to significantly influence key ecological processes such as nitrogen fixation. *Collema tenax* is the dominant N-fixing lichen in many BSCs, and the gross photosynthesis of this species was surprisingly found to be suppressed by the addition of nutrients such as P, K, and Zn [8]. It remains unclear, however, how nutrient amendment affects biogeochemical processes in the long term and how this may influence BSC stability [23].

For cyanobacterial BSCs, knowledge on the rapid cultivation through the direct broadcasting of crust samples remains scarce. The experimental study of multiple factors will be beneficial to realizing the rapid restoration of cyanobacterial crusts in the field. Based on crust sampling in the field, lab cultivation, and the investigation of biomass and surface changes, the present study examined the effects of light intensity, watering frequency, and nutrient amendment of N, P, K, and Ca on the development of cyanobacteria-dominated BSCs located in the Sonoran Desert in the southwestern United States. The results of this work improve our understanding of the factors that affect BSC development, and this information may narrow gaps between application and research to restore and engineer BSCs in arid and semiarid regions.

## Materials and Methods

### Soil Sampling and Preparation

Samples were collected from a plot regulated by Arizona State University and located in the Sonoran Desert near Phoenix, Arizona, USA (32°59′27′′N, 111°45′38′′W). Cyanobacterial crusts were collected from the soil surface (<10 mm depth), and the underlying soil was sampled from a depth >10 mm as the substrate. The crust sample and soil substrate were transported to the laboratory and sifted using a 5-mm sieve; the materials were then air dried and kept for use in subsequent experiments. This study was approved by the Department of Life Sciences, Arizona State University.

The crust sample was dominated by cyanobacteria, and no moss or lichen was observed. The dominant cyanobacteria at the end of the experiment was identified via microscopy as *Microcoleus vaginatus* Gom.

### Properties of the Crust and Soil Substrate

The initial chlorophyll *a* concentration of the BSC sample, determined using an acetone extraction method [24], was 1.28±0.08 µg/cm^2^, a value much lower than that of other well-developed cyanobacterial crusts (typically 10 to 50 µg/cm^2^, [25], [26]). The physical and chemical soil properties of both the crust and substrate samples were determined using standard methods [27], and the results are shown in Table 1.

**Table 1:** Selected physical and chemical properties of the soils of the study.

Sample	Particle size distribution			Total N (g kg^−1^)	Total P (g kg^−1^)	Total K (g kg^−1^)	CaCO_3_ (g kg^−1^)	TOC (%)	pH
	Sand (g kg^−1^)	Silt (g kg^−1^)	Clay (g kg^−1^)						
Crust	60.1	34.4	5.5	0.60	0.61	0.37	1.2	0.44	7.44
Substrate	72.5	23.3	4.2	0.20	0.44	0.30	1.2	0.16	7.72

### Experimental Design

Substrate soil (approximately 1,000 g) was encased in 15 cm×15 cm pots at a depth of 4.5 cm. Nutrient amendments (described below) were stirred into the crust sample, and the crust sample (approximately 200 g) was then homogenized and spread on top of the soil substrate to a depth of 0.5 cm in each pot.

Five external factors were evaluated: frequency of watering [5 or 10 day interval (W5 and W10, respectively)]; shade (S) or no shade (NS); NH_4_NO_3_ amendment (N) or no NH_4_NO_3_ amendment (NN); KH_2_PO_4_ amendment (KP) or no KH_2_PO_4_ amendment (NKP); and CaCO_3_ amendment (Ca) or no CaCO_3_ amendment (NCa). Full factorial combinations of these conditions were tested as described in Table 2. Three replicate growth experiments were performed for each treatment.

**Table 2:** Treatments used in the cyanobacteria-dominated crust cultivation experiments.

Treatment	W10																W5															
	S								NS								S								NS							
	N				NN				N				NN				N				NN				N				NN			
	KP		NKP		KP		NKP		KP		NKP		KP		NKP		KP		NKP		KP		NKP		KP		NKP		KP		NKP	
	Ca	NCa	Ca	NCa	Ca	NCa	Ca	NCa	Ca	NCa	Ca	NCa	Ca	NCa	Ca	NCa	Ca	NCa	Ca	NCa	Ca	NCa	Ca	NCa	Ca	NCa	Ca	NCa	Ca	NCa	Ca	NCa
Treatment No.	1	2	3	4	5	6	7	8	9	10	11	12	13	14	15	16	17	18	19	20	21	22	23	24	25	26	27	28	29	30	31	32
Combination	W10+S+N+KP+Ca	W10+S+N+KP	W10+S+N+Ca	W10+S+N	W10+S+KP+Ca	W10+S+KP	W10+S+Ca	W10+S	W10+N+KP+Ca	W10+ N+KP	1W10+N+Ca	W10+N	W10+KP+Ca	W10+KP	W10+Ca	W10	W5+S−N+KP+Ca	W5+S+N+KP	W5+S+N+Ca	W5+S+N	W5+S+KP+Ca	W5+S+KP	W5+S+Ca	W5+S	W5+N+KP+Ca	W5+N+KP	W5+N+Ca	W5+N	W5+KP+Ca	W5+KP	W5+Ca	W5

Note: W10∶300 ml deionized water was added to each sample every 10 days. W5∶300 ml deionized water was added to each sample every 5 days. S: each sample was covered by a 60% shade cloth at 20 cm above the pot. NS: no shading. N: 2.10 g NH_4_NO_3_ added to each pot. NN: no NH_4_NO_3_ added to any pots. KP: 1.05 g KH_2_PO_4_ added to each pot. NKP: no H_2_PO_4_ added to any pots. Ca: 2.10 g CaCO_3_ added to each pot. NCa: no CaCO_3_ added to any pots.

The treatment groups were watered every 5 or 10 days using a dripping system until each soil pot was saturated by 300 ml water. Double-deionized water was used to avoid the introduction of additional nutrients. A knitted black shade cloth, which excluded 60% of all incident light, was placed 20 cm above each pot in the shaded treatments. Nutrient amendments were supplied at the following levels: 2.1 g of NH_4_NO_3_ (300 kg/ha), 1.05 g of KH_2_PO_4_ (150 kg/ha), and 2.1 g of CaCO_3_ (300 kg/ha). Pots were incubated in a greenhouse with natural light for a total of 4 months, spanning April to August. The greenhouse was ventilated continuously and did not allow the incubating pots to be exposed to rain. The greenhouse temperatures fluctuated with the changing seasons.

### Indices and Data Analysis

The chlorophyll *a* content of the cyanobacteria-dominated BSCs was used as a proxy for their growth as biomass. At the end of the experiment, only the upper layer (≈ 4 mm) of the crust was used for the measurement of chlorophyll *a*. Exopolysaccharides (EPS, indicating the metabolic capacity of cyanobacterial crust) and soil organic matter (SOM) were measured using a combustion method and the phenol-sulfuric acid (PSA) test, respectively [27], [28]. Changes at the soil surface were recorded using a digital camera, and the crust coverage was measured using wire meshes with 100 grid squares (5×5 mm). Using the program SPSS 14.0, a paired-sample *t*-test (16 samples) was used to determine the significance of each factor with respect to the chlorophyll *a* concentration. The differences between each set of two factors were analyzed using a one-way ANOVA and LSD analysis (16 samples). The corresponding index is presented as the mean ± standard error.

## Results

### Changes in the Crust Surface Area

**Figure 1:**
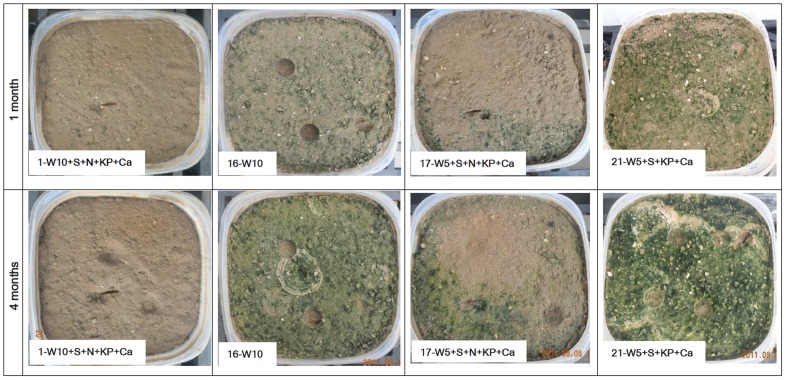
Visible changes in the soil surfaces after 1 and 4 months of cultivation in selected treatments.

Green surfaces indicative of photosynthetic pigments (e.g., chlorophyll *a*) were not present in any of the pots at the start of the experiment. However, the cyanobacterial crusts developed rapidly, as indicated by the development of green pigment after only 1 month. The coverage increased continually, and the surface color became darker over time, as shown in the photographs of the four treatment groups (treatments 1, 16, 17, and 21; Figure 1) which are the most representative of the surface differences. Figure 1 showed as well that, the treatment groups without NH_4_NO_3_ generally grew much faster than the other treatment groups. The treatment groups exposed to NH_4_NO_3_ developed patchy crusts, although additional watering produced better growth in these treatments [e.g., treatments 1 (W10+S+N+KP+Ca) and 17 (W5+S+N+KP+Ca)]. Watering every 5 days in combination with shade produced the best growth. The effects of CaCO_3_ and KH_2_PO_4_ on the cyanobacterial crusts were not significant based on the image analysis alone.

In addition to the visual illustration described above, the corresponding coverage of the cyanobacterial crust for those 4 treatments after 1 and 4 months was consistent with the photographic evidence. For example, in treatment groups 16 (less watering) and 21 (more watering), both without NH_4_NO_3_, the crust coverage reached 82.2±2.19% and 93.3±6.43%, respectively, within 1 month and increased to 95.7±1.86% and 98.3±0.33%, respectively, after 4 months. For treatment groups 1 (less watering) and 17 (more watering), both with NH_4_NO_3_, the crust coverage was 2.8±2.84% and 12.3±9.61%, respectively, after 1 month and increased to only 4.0±1.15% and 26.7±10.7%, respectively, after 4 months. These results indicated that the crust coverage of the treatment without NH_4_NO_3_ can reach high levels within a relatively short time, and additional watering enhanced this trend.

### Differences of Crust Biomass

**Figure 2:**
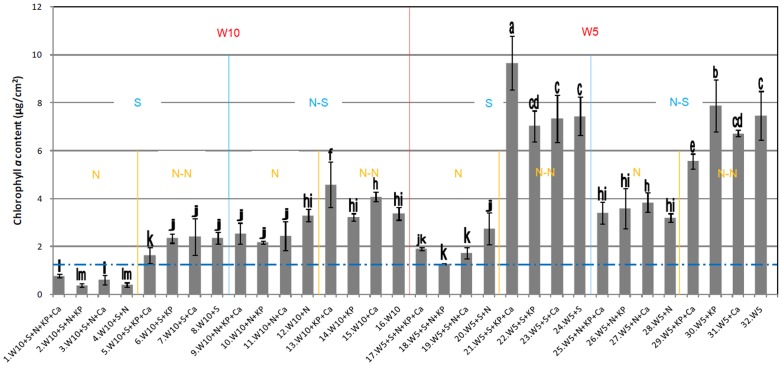
Chlorophyll *a* content in each of the 32 treatments after 4 months of cultivation. Note: W10∶300 ml deionized water was added to each sample every 10 days. W5∶300 ml deionized water was added to each sample every 5 days. S: each sample was covered by a 60% shade cloth positioned 20 cm above the pot. NS: no shading. N: 2.10 g NH_4_NO_3_ added to each pot. NN: no NH_4_NO_3_ added to any pots. KP: 1.05 g KH_2_PO_4_ added to each pot. NKP: no H_2_PO_4_ added to any pots. Ca: 2.10 g CaCO_3_ added to each pot. NCa: no CaCO_3_ added to any pots. The capital letters indicate the different treatments, while different lower case letters above the bars indicate significant differences between any two treatments at *P*<0.01.

Figure 2 shows the chlorophyll *a* levels of each treatment group after 4 months. The watering frequency, light intensity, and different nutrient amendments significantly influenced the crust development. The frequency of watering significantly increased the growth of crust biomass. The comparison of the treatment groups watered every 10 days (1–16) and the same treatments watered every 5 days (17–32) clearly indicated that a greater frequency of watering produced higher biomass. The highest biomass and best crust growth were produced under sufficient watering (treatments 21–24). However, the effects of watering on cyanobacterial growth varied with different the nutrient levels. The amendment of NH_4_NO_3_ inhibited the development of the cyanobacterial crust. In particular, the biomass levels of the crusts treated with NH_4_NO_3_ were consistently lower than those of the untreated crusts in the treatment groups that were watered every 5 days. The effects of KH_2_PO_4_ and CACO_3_ on the development of crusts were inconsistent; i.e., higher/lower biomass levels were observed in the presence/absence of both KH_2_PO_4_ and CACO_3_.

For the 5-day watering frequency treatments, the highest biomass was observed in the two shaded treatment groups [21 (W5+S+KP+Ca) and 23 (W5+S+Ca)]. This result indicated that shade could positively affect the development of crust biomass when the soil moisture content was high. However, the effects of shading were not consistent and often inhibited biomass accumulation. For example (Figure 2), shading led to the development of less crust biomass in the treatment groups watered every 10 days (treatment groups 1–16); this effect was more pronounced in the groups treated with NH_4_NO_3_ (1–4, 9–12). Shade inhibited crust growth in the treatment groups that received NH_4_NO_3_ (17–20, 25–28) but led to a slight enhancement of growth in the groups that were not treated with NH_4_NO_3_ (21–24, 29–32). Thus, the effect of shade may vary according to other factors, including growth stage, season, and availability of nutrients.

Further statistical analyses were performed to quantify the effects of the five factors on crust development. The paired-sample *t*-test showed that NH_4_NO_3_ and watering frequency had highly significant effects (*P*<0.01), whereas the degree of shading had a lower, although significant, effect on crust biomass development (*P*<0.05). CaCO_3_ and KH_2_PO_4_ had no significant effects on crust biomass development. The one-way ANOVA and LSD analysis indicated there was no significant difference between treatments that received NH_4_NO_3_ and the frequency of watering. Likewise, no significant difference was observed between treatments that received CaCO_3_ and KH_2_PO_4_. The effects of the five factors on cyanobacteria growth (in descending order of importance) were as follows: NH_4_NO_3_ ≈ watering frequency>shade>CaCO_3_ ≈ KH_2_PO_4_. The significant differences between all treatments (Figure 2) showed a similar trend as in the statistical analysis presented above.

### Crust Organic Matter and EPS

**Figure 3:**
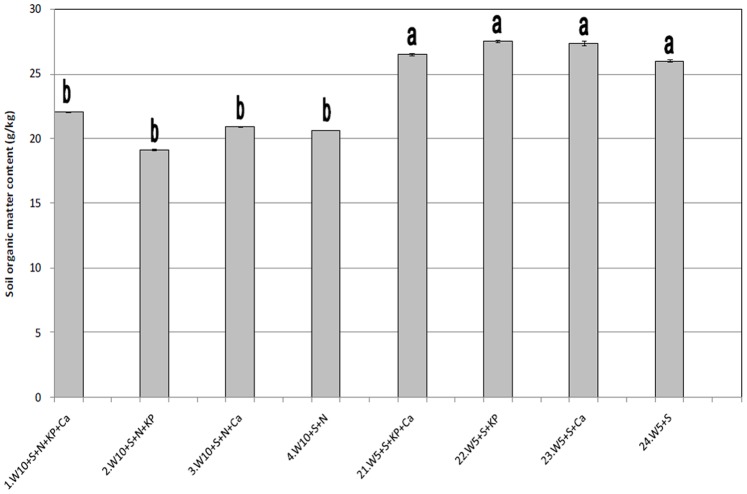
Soil organic matter content of the cyanobacterial crust layer in the treatments with the highest (21–24) and lowest (1–4) measured biomass. Note: Different letters above the bars indicate significant differences (*P*<0.01) between treatments.

**Figure 4:**
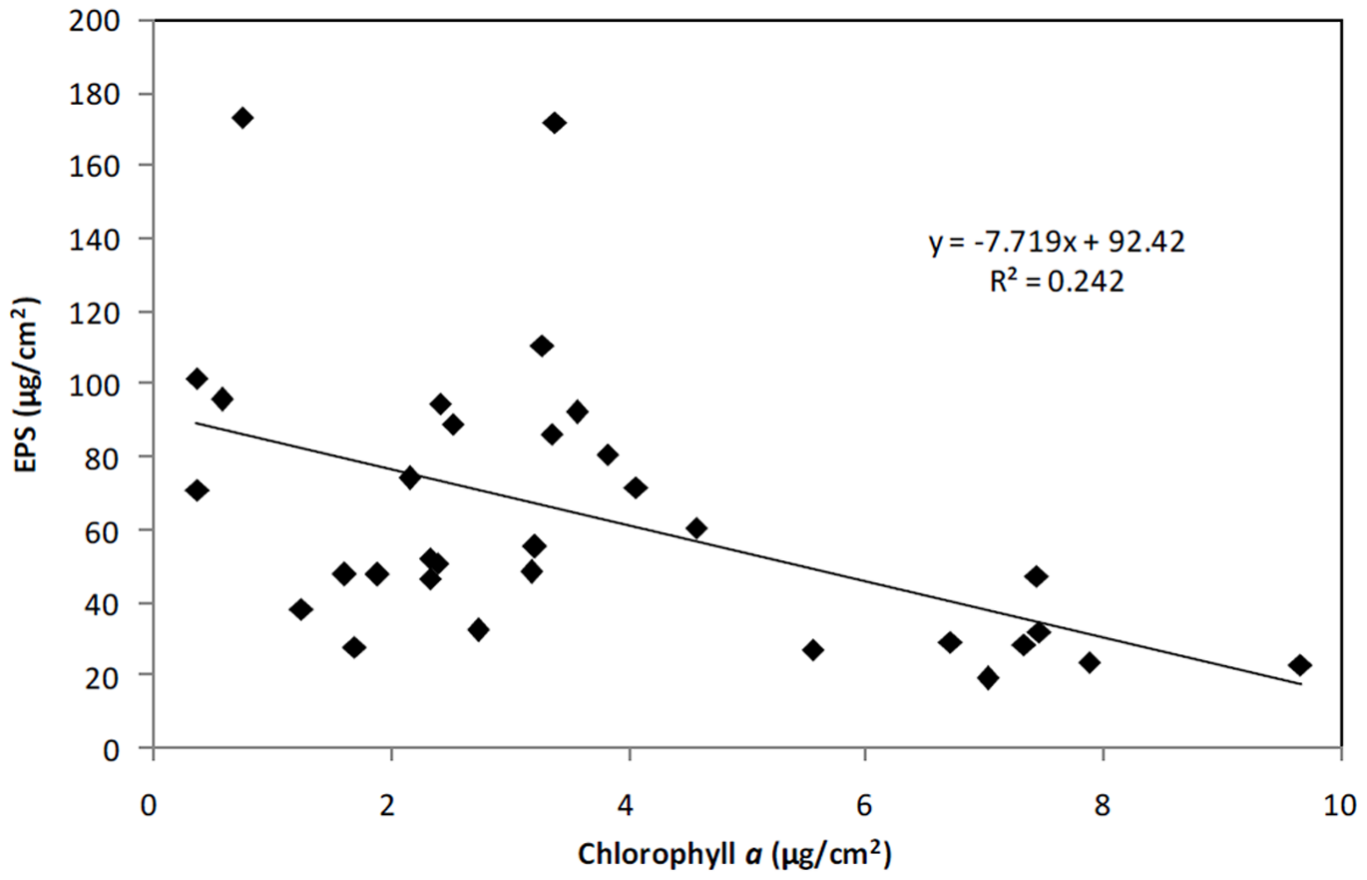
Relationship between chlorophyll *a* and exopolysaccharides (EPS).

To further examine the changes in crust development, the treatments with the lowest (treatments 1–4, watered every 10 days, not treated with NH_4_NO_3_) and highest (treatments 21–24, watered every 5 days, treated with NH_4_NO_3_) biomass levels were selected for the measurement of the soil organic matter in the cyanobacterial crust layer after 4 months of cultivation (Figure 3). This analysis indicated that the treatments with higher biomass levels contained more soil organic matter. We also measured the EPS contents as an indication of the metabolic capacity of the cyanobacterial crusts. The EPS contents of the crust layers that received the same treatments (Figure 4) were inversely proportional to their chlorophyll *a* contents and soil organic matter levels.

## Discussion

For a certain cyanobacterial crust, the external factors including the soil water content, availability of nutrients, light, and temperature are the main factors for rapid crust growth and development. Our results were both consistent and inconsistent with the findings of the previous studies. The reasons or interpretations behind these conclusions are discussed as follows.

### Nutrients Factor

In general, BSCs catalyze the fixation of CO_2_ and N_2_ and function to enrich the nutrient composition of soil [29], [30], [31], [32]. Thus, we assumed that nutrient amendment would facilitate the growth of BSCs, however, our results surprisingly contradicted this hypothesis. Specifically, the application of NH_4_NO_3_ inhibited the development of cyanobacterial crusts in our study, regardless of the soil moisture content or shade level, although BSCs are known to possess a high capacity for N_2_ fixation [33]. It was postulated that the disruption of the N_2_ fixation component of the niche inhibited the cyanobacteria growth. This result was in contrast to previous reports that most BSC types require significant amounts of C and N nutrients to maintain vigorous development [21], [34]. Furthermore, we found that higher K and Ca levels did not benefit the development of cyanobacterial crusts, which also contrasts with the results of a previous report [20]. This result may be due to the secondary roles of these nutrients in controlling crust development in comparison to the primary variables, such as light intensity and watering frequency. Clearly, these different results or abnormal phenomenon cannot be explained solely based on the current experiments. Further studies are warranted to determine how and why cyanobacterial crusts from different regions differentially respond to nutrient amendment, with the goal of developing a holistic understanding of the environmental conditions that promote optimal crust development.

### Soil Moisture Factor

The soil water content was found to represent a critical and positive factor for the development of BSCs in this study, a finding that is consistent with the results of previous studies. For example, the optimum moisture levels for net and gross photosynthesis in the cyanobacteria-dominated BSCs of China’s Loess Plateau were 40%–80% of the field water capacity [10]. Likewise, a study conducted in the Negev Desert indicated a strong positive relationship between the daylight wetness duration and the chlorophyll content of the crust, whereas the moisture content played an important role in stabilizing the BSC habitats [35]. As expected, our results were consistent with those of previous reports indicating that a higher soil water content or longer wetness duration significantly promotes the growth and development of cyanobacterial crust. Therefore, increasing the surface soil water and extending the wetness duration should be the most important mechanisms for rapidly restoring crusts in the field.

### Light and Temperature Factor

Previously, it was shown that the production of biomass was not linearly correlated with the temperature, light, or the availability of nutrients in the culture. Instead, the optimal biomass levels of *Microcoleus vaginatus* Gom. were produced when the temperature was 30°C ±2°C, the light was 600–700 µE/m^2^·s, and the renewal rate was 35% [19]. Zhao et al. [10] showed that the light compensation point and saturation point of a cyanobacteria-dominated BSC in the hilly Loess Plateau of China were <10 µmol/m^2^·s and 800 µmol/m^2^·s, respectively. In our study, light intensity also impacted the development of crust biomass. However, compared with previous reports focused on single factor, our experiment was conducted under variable temperature, light intensity, and soil water content conditions similar to the natural environment. For example, the ranges of light intensity with and without shading were 1.1–401.6 µmol/m^2^·s and 8.4–1164.3 µmol/m^2^·s, respectively on July 28^th^; meanwhile, the temperature fluctuated at 27.2–36.4°C and 27.8–39.4°C with and without shading, respectively, on July 28^th^. The total reduction of light associated with the shade cloth was 53.6%–86.9%, whereas the effect of shading on the temperature was minimal (0.6–3.0°C). Clearly, shading simultaneously changed the light intensity and temperature, which most likely resulted in actual temperature or light values that were lower than the optimal level on some days but higher on other days. For this reason, both inhibitory and promoting effects of shading were observed in the study, indicating that shading or the shading level in the field must be considered to obtain the optimal conditions.

### Relationship between Biomass and EPS

Unexpectedly, the EPS content was not positively correlated with the biomass level, as reported by a previous study [19]. We hypothesized that EPS might be consumed by heterotrophic organisms in the short term (during this period between the lasting watering event and EPS measurement, EPS was measured on the 5^th^ and 10^th^ day after the last watering event for the W5 and W10 treatments, respectively) or to produce filamentous cells of cyanobacteria in the soil crusts. These explanations would account for the higher biomass and lower EPS levels in these crusts. In addition, compared with studies conducted in the field [36], [37], our crust was cultivated in lab conditions and maintained for only a relatively short period (4 months), which may explain the negative relationship between the biomass and EPS. Further experimental studies are necessary to clearly address this question.

### Feasibility of Enhanced Restoration of Cyanobacterial Crusts

Wang et al. [16] and Rao et al. [38] both independently assessed the feasibility of using cyanobacterial culture inoculation to promote BSC formation in desert areas in Inner Mongolia. They found that crust coverage could reach 48.5% with an accompanying increase in the chlorophyll *a* content during the second year. However, they used a “culture inoculation” method experiencing isolation, purification, propagation and inoculation, which has a high cost and technical complexity. In this study, we attempted to use a “broadcasting” method through directly spreading the cyanobacterial crusts ‘diluted’ on the soil substrate and created the appropriate conditions for realizing rapid crust development, which is characterized by convenience and easy operation. The results showed that the spreading of cyanobacterial crust organisms onto a soil substrate with the appropriate regulation of the environment is also effective and feasible for the development of cyanobacteria-dominated BSCs. If effective techniques for condition regulation are developed, the current easy method of crust restoration could be widely applied and engineered in the field.

## Conclusions

Our study demonstrated that the cultivation and development of cyanobacteria-dominated crusts through a “broadcasting” method is feasible. Soil moisture is a primary factor influencing the growth of cyanobacteria and significantly promoted the development of cyanobacteria-dominated BSCs. In contrast, amendment with NH_4_NO_3_ had significant negative effects on the growth of cyanobacterial crusts in the current experiment, whereas KH_2_PO_4_ and CaCO_3_ had nonsignificant effects on growth. Shading may positively or negatively impact the crust growth depending on whether the temperature and light intensity under shading are lower or higher than the required optimal condition. Five factors impact BSC growth with the following order of importance: NH_4_NO_3_≈watering frequency>shading>CaCO_3_≈KH_2_PO_4_. The specific reason for the negative correlation between EPS and biomass should be studied in further research. In the field, we suggested that maintaining high soil moisture content by reducing runoff and increasing infiltration facilitates the growth and restoration of cyanobacterial crusts, meanwhile fertilization with NH_4_NO_3_ should be avoided. Our understanding of the factors that influence BSC development will facilitate studies regarding the restoration and cultivation of BSCs.

## References

[1] Belnap J (2003) The world at your feet: desert biological soil crusts. Front Ecol Environ 1: 181–189.

[2] Meeting B (1991) Biological surface features of semiarid lands and deserts. In: Skujins J, editor. Semiarid and sand deserts: soil resource and Reclamation. New York: Marcel Dekker. 257–293.

[3] Tighe M, Haling RE, Flavel RJ, Young IM (2012) Ecological succession, hydrology and carbon acquisition of biological soil crusts measured at the micro-scale. PLoS ONE 7(10): e48565 doi:10.1371/journal.pone.0048565 23119058 10.1371/journal.pone.0048565 PMC3484118

[4] Angel R, Matthies D, Conrad R (2011) Activation of methanogenesis in arid biological soil crusts despite the presence of oxygen. PLoS ONE 6(5): e20453 doi:10.1371/journal.pone.0020453 21655270 10.1371/journal.pone.0020453 PMC3105065

[5] Bowker MA, Maestre FT, Escolar C (2009) Biological crusts as a model system for examining the biodiversity ecosystem function relationship in soils. Soil Biol Biochem 42: 1–13.

[6] Bu CF, Cai GQ, Zhang XC, Ma L (2008) Review on developmental characteristics and ecological functions of soil crust. Prog Geogr 27: 26–31 (in Chinese with English abstract)..

[7] Bowker MA, Belnap J, Chaudhary VB, Johnson NC (2008) Revisiting classic water erosion models in drylands: The strong impact of biological soil crusts. Soil Biol Biochem 40: 2309–2316.

[8] Zhang YM, Wang HL, Wang XQ, Yang WK, Zhang DY (2006) The microstructure of microbiotic crust and its influence on wind erosion for a sandy soil surface in the Gurbantunggut Desert of Northwestern China. Geoderma 132: 441–449.

[9] Zhao YG, Xu MX, Belnap J (2010) Response of biocrusts’ photosynthesis to environmental factors: a possible explanation of the spatial distribution of biocrusts in the Hilly Loess Plateau region of China. Acta Ecol Sin 30: 4668–4675 (in Chinese with English abstract)..

[10] Bu CF, Wu SF, Yang YS, Han FP, Meng J (2013) Interactive effects of moss-dominated biological soil crusts and different types of vegetation on erosion and soil water. Soil Use Manage Under review.

[11] Wang XQ, Zhang YM, Zhang WM, Han ZW (2004) Wind tunnel experiment of biological crust effect on wind erodibility of sand surface in gurbantunggut desert, Xinjiang. J Glaciol Geocryol 26: 632–638.

[12] Wei JC (2005) Biocarpet engineering using microbiotic crust for controlling sand. Arid Zone Res 22: 287–288 (in Chinese with English abstract)..

[13] Hu CX, Liu YD, Song LR, Zhang DL (2002) Effect of desert soil algae on the stabilization of fine sands. J Appl Phycol 14: 281–292 (in Chinese with English abstract)..

[14] Rao BQ, Liu YD, Hu CX, Li DH, Shen YW (2009) The technology of man-made algal crust and its applications in control of desertification. Acta Hydrobiol Sin 33: 756–761 (in Chinese with English abstract)..

[15] Wang WB, Liu YD, Li DH, Hu CX, Rao BQ (2009) Feasibility of cyanobacteria inoculation for biological soil crusts formation in desert area. Soil Biol Biochem 41: 926–929.

[16] Tang DS, Wang WB, Li DH, Hu CX, Liu YD (2009) Effects of artificial algal crust on soil enzyme activities of Hopq desert, China. Acta Hydrobiol Sin 31: 339–344 (in Chinese with English abstract)..

[17] Xie ZM, Liu YD, Hu CX, Chen LZ, Li DH (2007) Relationships between the biomass of algal crusts in fields and their compressive strength. Soil Biol Biochem 39: 567–572.

[18] Bu CF, Wu SF, Zhang KK, Yang YS, Gao GX (2013). Biological Soil Crusts: an Eco-adaptive Biological Conservative Mechanism and Implications for Ecological Restoration. Plant Biosyst DOI:10.1080/11263504.2013.819820.

[19] Xie ZM, Liu YD, Chen LZ, Hu CX, Li DH, et al (2008) The effects of different cultivation conditions on the biomass and exopolysaccharide production by *Microcoleus Vaginatus* Gom. Acta Hydrobiol Sin 32: 272–275 (in Chinese with English abstract)..

[20] Li XR, He MZ, Zerbe S, Li XJ, Liu LC (2010) Micro-geomorphology determines community structure of biological soil crusts at small scales. Earth Surf Proc Land 35: 932–940.

[21] Maestre FT, Martín N, Díez B, Lopez-Poma R, Santos F, et al (2006) Watering, fertilization, and slurry inoculation promote recovery of biological crust function in degraded doils. Microbial Ecol 52: 365–377. 10.1007/s00248-006-9017-0 16710791

[22] Wu H, Wang HL, Liang SM, Nie HL, Zhang YM (2006) Temporal-spatial dynamics of distribution patterns of microorganism relating to biological soil crusts in the Gurbantunggut Desert. Chinese Sci Bull 51: 124–131.

[23] Bu CF, Wu SF, Xie YS, Zhang XC (2013) The Study of Biological Soil Crusts: Hotspots and Prospects. CLEAN: Soil Air Water 41(9): 899–906.

[24] Tsujimura S, Nakahara H, Ishida N (2000) Estimation of soil algal biomass in salinized irrigation land: a comparison of culture dilution and chlorophyll a extraction methods. J App Phycol 12: 1–8.

[25] Bowker MA, Reed SC, Belnap J, Phillips SL (2002) Temporal variation in community composition, pigmentation, and Fv/Fm of desert cyanobacteria soil crusts. Microbial Ecol 43: 13–25. 10.1007/s00248-001-1013-9 11984625

[26] Lan SB, Wu L, Zhang DL, Hu CX, Liu YD (2011) Ethanol outperforms multiple solvents in the extraction of chlorophyll-*a* from biological soil crusts. Soil Biol Biochem 43: 857–861.

[27] Institute of Soil Science, Chinese Academic Sciences (1978) Analysis of physical and chemical properties of soil. Shanghai: Shanghai Science and Technology Press. (in Chinese).

[28] Dubois M, Gilles KA, Hamilton JK, Rebers PT, Smith F (1956) Colorimetric method for determination of sugars and related substances. Anal Chem 28: 350–356.

[29] Belnap J, Harper KT, Warren SD (1994) Surface disturbance of cryptobiotic soil crusts: Nitrogenase activity, chlorophyll content, and chlorophyll degradation. Arid Soil Res Rehab 8: 1–8.

[30] Evans RD, Johansen JR (1999) Microbiotic crusts and ecosystem processes. Crit Rev Plant Sci 18: 183–225.

[31] Guo YR, Zhao HL, Zuo XA, Sam D, Zhao XY (2008) Biological soil crust development and its topsoil properties in the process of dune stabilization, Inner Mongolia, China. Environ Geol 54: 653–662.

[32] Wu N, Zhang YM, Downing A (2009) Comparative study of nitrogenase activity in different types of biological soil crusts in the Gurbantunggut Desert, Northwestern China. J Arid Environ 73: 828–833.

[33] Housman DC, Powers HH, Collins AD, Belnap J (2006) Carbon and nitrogen fixation differ between successional stages of biological soil crusts in the Colorado Plateau and Chihuahuan Desert. J Arid Environ 66: 620–634.

[34] Chen YQ, Zhao YG, Ran MY (2011) Influence of 4 nutrients on the development of moss crust. J Northwest A&F Univ (Nat. Sci. Ed.) 39(5): 44–50 (in Chinese with English abstract)..

[35] Kidron GJ, Vonshak A, Abeliovich A (2009) Microbiotic crusts as biomarkers for surface stability and wetness duration in the Negev Desert. Earth Surf Process Land 34: 1594–1604.

[36] Chen LZ, Liu YD, Song LR (2002) The function of exopolysaccharides of *Microcoleus* in the formation of desert soil. Acta Hydrobiol Sin 26(2): 155–159.

[37] De Philippis R, Vincenzini M (2003) Outermost polysaccharidic investments of cyanobacteria: Nature, significance and possible applications. Recent Res Devel Microbiol 7: 13–22.

[38] Rao BQ, Wang WB, Lan SB, Li DH, Hu CX (2009) Development characteristics and distribution of microorganisms within 3-year-old artificial algal crusts in Hopq desert. Acta Hydrobiol Sin 33: 937–944 (in Chinese with English abstract)..

